# FORC-Diagram Analysis for a Step-like Magnetization Reversal in Nanopatterned Stripe Array

**DOI:** 10.3390/ma14247523

**Published:** 2021-12-08

**Authors:** Victor K. Belyaev, Dmitry Murzin, Jose C. Martínez-García, Montserrat Rivas, Nikolay V. Andreev, Aleksei G. Kozlov, Aleksei Yu. Samardak, Alexey V. Ognev, Alexander S. Samardak, Valeria Rodionova

**Affiliations:** 1 The Sophia Kovalevskaya North-West Center for Mathematical Research Center, Immanuel Kant Baltic Federal University, 236016 Kaliningrad, Russia; DMurzin1@kantiana.ru (D.M.); VVRodionova@kantiana.ru (V.R.); 2 Research and Education Center “Smart Materials and Biomedical Applications”, Institute of Physics, Mathematics and Information Technology, Immanuel Kant Baltic Federal University, 236001 Kaliningrad, Russia; 3 Department of Physics & IUTA, Universidad de Oviedo, 33204 Gijon, Spain; jcmg@uniovi.es (J.C.M.-G.); rivas@uniovi.es (M.R.); 4 Materials Science and Metallurgy Shared Use Research and Development Center, National University of Science and Technology MISiS, 119049 Moscow, Russia; andreevn.misa@gmail.com; 5 Laboratory of Spin-Orbitronics, Institute of High Technologies and Advanced Materials, Far Eastern Federal University, 690922 Vladivostok, Russia; kozlov.ag@dvfu.ru (A.G.K.); lsamardak@gmail.com (A.Y.S.); ognev.av@dvfu.ru (A.V.O.); samardak.as@dvfu.ru (A.S.S.)

**Keywords:** magnetic anisotropy, magnetic microstructure, simulation, FORC-diagram analysis

## Abstract

The fabrication approach of a magnonic crystal with a step-like hysteresis behavior based on a uniform non-monotonous iron layer made by shadow deposition on a preconfigured substrate is reported. The origin of the step-like hysteresis loop behavior is studied with local and integral magnetometry methods, including First-Order Reversal Curves (FORC) diagram analysis, accompanied with magnetic microstructure dynamics measurements. The results are validated with macroscopic magnetic properties and micromagnetic simulations using the intrinsic switching field distribution model. The proposed fabrication method can be used to produce magnonic structures with the controllable hysteresis plateau region’s field position and width that can be used to control the magnonic crystal’s band structure by changing of an external magnetic field.

## 1. Introduction

One of the promising candidates for replacing conventional semiconductor electronics are spin-wave-based devices [1,2,3]. The field of science that studies signal generation and transport with spin waves is called magnonics. In magnonics, spin waves are considered as a collective spin oscillation in a magnetic medium similar to plasmon-polaritons in plasmonics [4]. Compared to CMOS-based electronics, magnonics can provide low-cost nanoscale room temperature signal processing at GHz frequencies still compatible with CMOS-based devices in spin-wave CMOS systems [1]. Still, a magnonic device for signal processing requires a special type of waveguide capable of transporting and transforming spin waves at the nanoscale—magnonic crystals [5]. Periodical variation of the magnitude and phase of spin waves in magnonic crystals results in the presence of a bandgap structure that prohibits the transportation of certain spin waves [6,7]. This makes magnonic crystals competitive in fields of logic, quantum, and optoelectronic computing along with plasmonics, photonics, and phononics. A comprehensive up-to-date review on the most important applications of magnonics has recently been reported in [8].

The magnonic crystal band structure can be tuned up by using ferromagnetic structures exhibiting strong magnetic interactions leading to a step-like hysteresis behavior [7,9,10]. In such systems, the frequency and velocity of the spin wave modes can be shifted up or down depending on the intermediate magnetization states of the magnonic crystal. Step-like hysteresis can be achieved in ferromagnetic films with surface roughness modulation [11,12,13] or by altering magnetic domains separated by anisotropy-constrained domain walls [14,15], usually made by top-down and bottom-up approaches. Top-down approaches, such as UV lithography, e-beam lithography, and chemical etching, are used to fabricate well-defined nanostructures with high-quality interfaces to adjust the magnon propagation band structure and study of spin wave interactions in non-trivial spin textures. The bottom-up approaches, like two-photon lithography or thermally assisted magnetic scanning probe lithography, are used to create nanoscale spin textures with unique magnetic microstructure, topology, and reconfigurable spin-wave channels.

The alternative approach to making a magnonic crystal with step-like hysteresis behavior is the deposition of ferromagnetic material on the top of a preconfigured polymer substrate or superlattice with stripes made by printing or stamping [16]. The thickness of the ferromagnetic material layer on different sections of the stripes is adjusted by using the ‘shadow deposition’ effect. This approach can be used to make structures with a plateau region in a step-like hysteresis loop in a single deposition cycle. In the case of iron layer deposition, this is achieved if the layer thickness is comparable to or smaller than the height of the stripes [17]. To tune the plateau width and field position, it is important to understand the origin of the magnetic phases and interactions within the structure.

In this work, Kerr magnetometry, magnetic force microscopy and first-order reversal curves (FORC) diagram analysis of experimental and simulated data are used to describe magnetic interactions and step-like magnetization reversal of a magnonic crystal based on a thin polycrystalline iron layer.

## 2. Materials and Methods

The sample was made of consistently formed layers of 100 nm silver, 5 nm iron, and 20 nm silica nitride on top of a polymer substrate with trapezoidal stripes by the ion-beam deposition method. The silver and silica nitride layers protected the iron layer from degradation. The deposition was done at normal incidence. During deposition, the substrate holder was rotated with a constant angular speed of 45 rpm. The parameters of stripe arrays were characterized with the NT-MDT Integra Aura atomic force microscope in the semi-contact mode and the scanning electron microscope Hitachi NB5000 combined with a focused ion beam. The period and height of the stripes were equal to 740 nm and 100 nm. The edges of the stripes were tilted at the angle of 50° with respect to the plane of the substrate [17].

**Figure 1:**
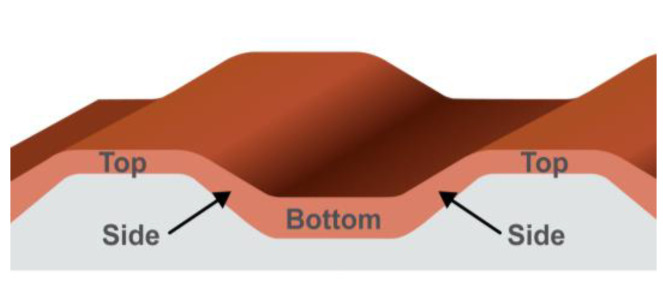
Schematic image of an iron layer on a substrate period.

Each stripe period had a bottom part, two sides, and a top part. The width and thickness of the iron layer were verified with the JEM 2100 transmission electron microscope by JEOL. The schematic representation of the iron layer is demonstrated in Figure 1. The iron layer was continuous and had non-uniform thickness across the profile. The widths of the parts and the corresponding iron thicknesses are shown in Table 1.

Integral and local measurements of magnetization reversal were done with the LakeShore 7404 vibrating sample magnetometer and NanoMOKE II scanning laser magnetometer in the geometry of longitudinal Kerr effect, respectively. The vibrating sample magnetometer has a noise floor of 10^−6^ emu at 3 s/pt; the NanoMOKE II has a sensitivity of 0.5 mdeg (rms) at an excitation frequency of 13 Hz with a light beam focused in a 10 um^2^ spot.

The magnetic microstructure and dynamics of the sample were studied with the magnetic force microscope (MFM) using the TipsNano magnetic cantilever MFM01. Demagnetization of the sample was done along the stripes in the AC magnetic field with the frequency of 18 Hz. The magnetic microstructure dynamics study was performed on a fixed region of the sample’s surface in several steps. First, the negative saturating field was applied. Then, it was switched off and an image of the magnetic microstructure in the remanent magnetization state *M_r_* was obtained. Before each subsequent measurement a DC magnetic field with a gradually increased magnitude from $M_{r}$ at 0 mT to the positive saturation $M_{s}$ at 20 mT was applied to the sample. All MFM measurements were made at a distance of 100 nm between the surface of the sample and the cantilever. The magnetic microstructure dynamics study was done only for the top parts of the sample stripes without shifting the domain walls with the cantilever stray fields.

The vibrating sample magnetometer was used to obtain a set of first-order reversal curves (FORCs) for distinguishing magnetic contributions from the magnetic phases. The measurement of a FORC starts by applying a saturating magnetic field $H_{sat}$ aligned parallel to the stripes, which is reduced to a reversal field $H_{r}$; from this value, the field is increased back to the $H_{sat}$ while measuring the magnetization $M\left(H,H_{r}\right)$ with the field step $H_{step}$. Therefore, all measured data consists of *M*-*H* minor loops starting at $H_{r}$ and ending at positive saturation $H_{sat}$. The procedure for getting the so-called FORC diagram requires repeating the described step for a set of decreasing equispaced values of $H_{r}$ until reaching the negative saturation. Obtained data by this way allowed us to calculate the FORC diagram in two steps: First, we calculated the switching field distribution (SFD) of each curve defined by its return field $H_{r}$:

(1) $$\mathrm{SFD}\left(H,H_{r}\right)={\left(\frac{\partial M}{\partial H}\right)}_{H_{r}}$$

Then, we obtained the FORC density as the mixed derivative:

(2) $$\rho \left(H,H_{r}\right)=-\frac{\partial \mathrm{SFD}\left(H.H_{r}\right)}{\partial H_{r}}=-\frac{\partial^{2}M\left(H,H_{r}\right)}{\partial H_{r}\partial H}$$

The bidimensional plot of $\rho \left(H,H_{r}\right)$ is the FORC diagram [18]. For comparison with the Preisach plane, a change of variables is applied:

(3) $$H_{c}=\left(H-H_{r}\right)/2$$

(4) $$H_{u}=\left(H+H_{r}\right)/2$$

It is well known that numerical differentiation can enormously degrade the signal-to-noise ratio because the noise will be amplified in proportion to its frequency, which means that high-frequency spurious signals may acquire undesired importance to the extent of blurring or hiding essential information features of the meaningful signal. gFORC software performs the differentiation on their discrete Fourier transform to cope with this problem using Equations (1) and (2). Differentiating in the frequency-domain reduces then to a simple operation of the Fourier coefficients followed by the inverse Fourier transform operation to get the derivatives in the time domain [19].

## 3. Results and Discussions

**Figure 2:**
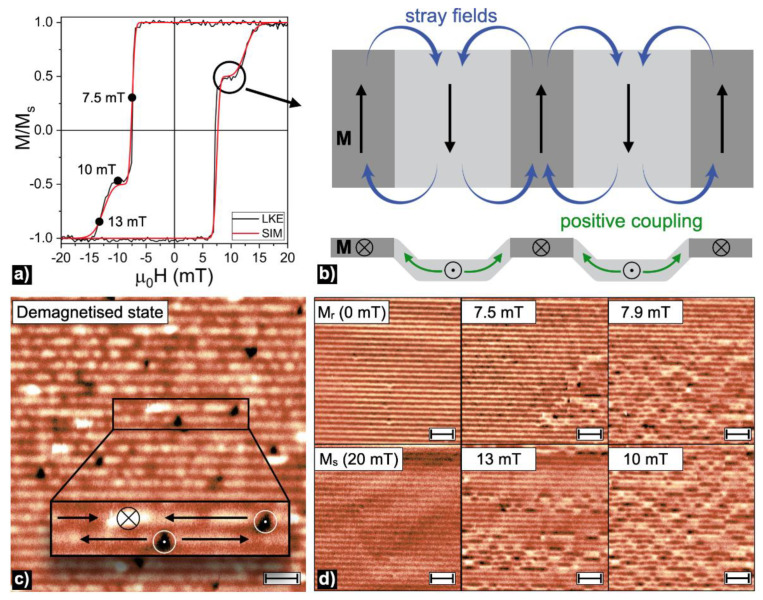
(**a**) Local and simulated hysteresis loops along the EMA direction. (**b**) The schematic image of the structure’s bottom, side and top parts’ magnetization distribution in the hysteresis loop’s plateau region. Arrows show the magnetization direction M (black), positive coupling (green) between bottom and side parts, and stray field (blue) delaying magnetization reversal of the top parts. The MFM images of magnetic microstructure in (**c**) the demagnetized state, the scale bar is 2 μm, and (**d**) magnetic microstructure dynamics with the scale bars of 5 μm. Arrows in the inset of panel (**c**) indicate an orientation of magnetization components at a given area on the top part of the stripes.

The sample had a geometry-driven anisotropy of magnetic properties that formed an in-plane easy magnetization axis (EMA) parallel to the stripes and a hard magnetization axis perpendicular to them, in accordance with similar structures [17,20]. All magnetic measurements were made along the EMA direction. Experimental and simulated hysteresis loops, as well as the images of the magnetic microstructure in the demagnetized state and during the magnetization dynamics measurements, are shown in Figure 2.

The experimentally obtained hysteresis loop had a step-like behavior indicating the presence of at least two magnetic phases. Observed switching fields of $\mu_{0}H_{C1}=7.2\;\mathrm{mT}$ and $\mu_{0}H_{C2}=12.7\;\mathrm{mT}$ corresponded to the 75% and 25% of sample’s magnetic moment change, respectively. These changes are in agreement with the magnetization reversal of iron volume deposited on the bottom and side parts of the period followed by the switching of the iron covering the top parts (Table 1). To estimate the contribution of the different parts of the structure to the hysteresis loop, macromagnetic simulations were performed as in [21]. The simulation accounts for reversible and irreversible magnetization reversals by creating a SFD that results from combining a Gaussian function g(H) (corresponding to reversible processes) and a log-normal one L(H) (for the irreversible processes):

(5) $$g\left(H\right)=\frac{1}{\sigma_{r}\sqrt{2\pi }}\exp\left[-\frac{1}{2}{\left(\frac{H}{\sigma_{r}}\right)}^{2}\right]$$

(6) $$L\left(H\right)=\frac{1}{\sqrt{2\pi }\sigma_{i}H}\exp\left(-\frac{1}{2\sigma_{i}^{2}}{\left[\ln\frac{H}{H_{C}}\right]}^{2}\right)$$

where H is the applied magnetic field, $H_{C}\exp\left(\sigma_{i}^{2}\right)$ is the mean switching field, $\sigma_{r}$ and $\sigma_{i}$ are the standard deviations of the reversible and irreversible switching field distributions.

Magnetic moments behaved in reversible and irreversible ways depending on the proximity of their orientation to the EMA, as can be well understood by recalling the Stoner–Wohlfarth model. For this reason, in the hysteresis loop simulations carried out for this work, a global SFD has been chosen in which the reversible and irreversible behaviors are combined magnetic moment-to-magnetic moment through a convolution of both reversible and irreversible SFDs:

(7) $$\mathrm{SFD}\;\left(H\right)=g\left(H\right)*L\left(H\right)=\overset{\infty }{\underset{-\infty }{\int }}g\left(\eta \right)*L\left(H-\eta \right)d\eta$$

Then, different parts of the sample with different magnetic properties were additively combined. To obtain the hysteresis loops, we integrated the SFD(H) in H from the maximum to the minimum applied field. According to the simulation shown in Figure 2a, 75% of the material had a coercive field distribution around $H_{C1}=6\;\mathrm{kA}/m\;\left(\mu_{0}H_{C1}=7.2\;\mathrm{mT}\right)$ with $\sigma_{r}=4$ and $\sigma_{i}=0.02$; the other 25% of the material had a coercive field distribution centered at $H_{C2}=10\;\mathrm{kA}/m\;\left(\mu_{0}H_{C2}=12.7\;\mathrm{mT}\right)$, with $\sigma_{rev}=2$ and $\sigma_{irr}=0.11$.

Images of the magnetic microstructure had additional non-magnetic stripe-like contrast, clearly seen in the Figure 2d for $M_{r}$ and $M_{s}$ states. The distance from the MFM cantilever to the structure’s top and bottom parts was fixed to the 100 nm and 200 nm, respectively. This difference was resulted in the MFM sensitivity only to the top parts’ magnetization dynamics. Bright white and black spots in Figure 2c,d were related to the presence of out-of-plane magnetic moments [22]. In the demagnetized state, each top part contained alternating white and black spots indicating the presence of domains with in-plane magnetic moments separated by 180° Neel domain walls (inset in Figure 2c) [23,24,25].

These results can be interpreted as a magnetization reversal along the EMA direction taking place in two steps. The coercive force of the iron film increases with decreasing layer thickness [17,24]. Stripes’ bottom parts were covered with the thickest layer of iron and switched in the field of 7.5 mT. The iron deposited on the side and bottom parts of the stripes was ferromagnetically coupled and switched the magnetization at the same field. The magnetization switching of the bottom and side parts caused a demagnetization field, delaying the magnetization reversal of the top parts and producing the curve’s plateau from 8.1 mT to 10.7 mT. Schematically this magnetization state is demonstrated in Figure 2b. The second step was the magnetization reversal of the top parts of the structure. According to the volume of iron deposited in the top parts of the structure (Table 1), it corresponded to the switch of 25% of the iron volume in the field of 12.5 mT (Figure 2a).

**Figure 3:**
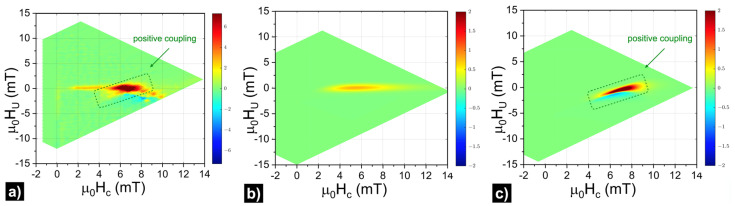
(**a**) The experimentally obtained FORC diagram and simulated FORC diagrams for (**b**) non-interacting and (**c**) positively coupled magnetic systems.

The FORC-diagram technique was used to study the magnetic interactions in the sample. Integral data from the bulk sample was collected with the VSM, so an additional coercive field distribution centered below 7.5 mT with a large dispersion in its values was observed. It can be assigned to the sample borders. The experimental and simulated FORC diagrams are shown in Figure 3.

The experimental FORC diagram (Figure 3a) was plotted with an optimal Gaussian filter width σ=0.2 mT, equivalent to a smoothing factor of 1.55 in the classical fitting algorithm [18]. The resulting FORC-diagram pattern had a complex shape that can be simplified as the superposition of two fingerprints. To clarify this explanation, two FORC diagrams were simulated: one corresponding to a set of non-interacting magnetic defects (Figure 3b) and one made of a ferromagnetically coupled phase corresponding to the interactions between iron deposited onto bottom and side parts (Figure 3c).

The simulated FORC diagrams shown in panels (b) and (c) of Figure 3 were obtained considering two contributions. The first comes from defects at the sample borders, while the second comes from the bottom and side parts of the nanostructure. Correspondingly, two distributions of switching fields were used. One centered at $\mu_{0}H_{C0}=6.4\;\mathrm{mT}$ ($\sigma_{r}=10$, $\sigma_{i}=0.4$) with no magnetic interaction, to account for isolated defects at the sample borders. As can be seen in Figure 3b, the fingerprint of a non-interacting magnetic system is a spot of positive 𝜌 values elongated along the horizontal axis ($H_{U}=H_{C}$). Its length was associated with the distribution width. The other switching field distribution, centered at $\mu_{0}H_{c1}=7.5\;\mathrm{mT}$ ($\sigma_{r}=15$, $\sigma_{i}=0.2$), represents the bottom and side parts of the nanostructure. A magnetizing interaction was considered following a mean-field model in which, at each point of the curve, an interaction field $kM/M_{S}\;$ is defined by the interaction constant k. In the presented simulation, k was 1.2 mT. The characteristic pattern of positively coupled magnetic systems presented a boomerang shape with yellow-red colors on top of it (positive values) and blue at the bottom (negative values). The superposition of both patterns had a remarkable resemblance to the experimental pattern as shown in Figure 3a. Similar simulation results were obtained in previous works [21,26]. On the right side of the boomerang, there were red-blue pairs of spots related to slightly different coercive values on various parts of the stripe structure. The contribution of the stripe array’s top parts with the switching field of 12.5 mT falls out of the range of applied fields to measure the FORCs, preventing it from being included in this analysis. In any case, the fact that these parts switch at a different field, indicates a weakly coupling to the bottom and side parts.

## 4. Conclusions

A way to achieve a step-like hysteresis loop by deposition of a single homogeneous iron layer with nonmonotonic thickness on the top of a preconfigured polymer substrate was demonstrated. The interactions between magnetic phases in the sample were studied with FORC-diagram analysis of the experimental and simulated data, having good resemblance with results of macroscopic magnetic properties simulations. These approaches assisted with the study of magnetic microstructure dynamics were used to describe a sequence of magnetization reversal and to explain the origin of separate magnetic phases. The step-like behavior of a hysteresis loop was due to the magnetostatic interaction between the structure’s bottom and side parts and the top parts. According to the results, it is possible to control the field position and width of the plateau region of hysteresis by changing the thickness of iron in different parts of the structure or the period, width, and height of the stripes.

## Figures and Tables

**Table 1:** The width and corresponding thicknesses of the iron layer obtained by TEM images. The iron volume values correspond to the amount of iron in each separate part with respect to the total iron volume deposited into the single period of a structure.

Parameter/Part Name	Top	Sides	Bottom
Part width (nm)	236 ± 7	146 ± 4	212 ± 6
Iron layer thickness (nm)	8 ± 1	10 ± 3	20 ± 3.2
Iron volume (%)	24 ± 2.4	40 ± 6.2	36 ± 5.4

## Data Availability

Not applicable.
